# Endoplasmic Reticulum Stress-Associated Lipid Droplet Formation and Type II Diabetes

**DOI:** 10.1155/2012/247275

**Published:** 2012-02-28

**Authors:** Xuebao Zhang, Kezhong Zhang

**Affiliations:** ^1^Center for Molecular Medicine and Genetics, The Wayne State University School of Medicine, 540 East Canfield Avenue, Detroit, MI 48201, USA; ^2^Department of Immunology and Microbiology, The Wayne State University School of Medicine, Detroit, MI 48201, USA; ^3^Karmanos Cancer Institute, The Wayne State University School of Medicine, Detroit, MI 48201, USA

## Abstract

Diabetes mellitus (DM), a metabolic disorder characterized by hyperglycemia, is caused by insufficient insulin production due to excessive loss of pancreatic *β* cells (type I diabetes) or impaired insulin signaling due to peripheral insulin resistance (type II diabetes). Pancreatic *β* cell is the only insulin-secreting cell type that has highly developed endoplasmic reticulum (ER) to cope with high demands of insulin synthesis and secretion. Therefore, ER homeostasis is crucial to the proper function of insulin signaling. Accumulating evidence suggests that deleterious ER stress and excessive intracellular lipids in nonadipose tissues, such as myocyte, cardiomyocyte, and hepatocyte, cause pancreatic *β*-cell dysfunction and peripheral insulin resistance, leading to type II diabetes. The excessive deposition of lipid droplets (LDs) in specialized cell types, such as adipocytes, hepatocytes, and macrophages, has been found as a hallmark in ER stress-associated metabolic diseases, including obesity, diabetes, fatty liver disease, and atherosclerosis. However, much work remains to be done in understanding the mechanism by which ER stress response regulates LD formation and the pathophysiologic role of ER stress-associated LD in metabolic disease. This paper briefly summarizes the recent advances in ER stress-associated LD formation and its involvement in type II diabetes.

## 1. Introduction to ER Stress

ER is an intracellular organelle where dynamic protein folding and assembly, storing cellular calcium, and lipid biosynthesis occur. A variety of biochemical or pathophysiological stimuli can interrupt protein folding process in the ER by disrupting protein glycosylation, disulfide bond formation, or ER calcium pool. These disruptions can cause the accumulation of unfolded or misfolded proteins in the ER lumen, a condition termed as “ER stress” [1, 2]. To protect cells from proteotoxicity caused by ER stress, the unfolded protein response (UPR) is activated through attenuating general protein translation, increasing in protein folding capacity, and expediting degradation of misfolded proteins. Three major ER stress sensors or transducers have been found: inositol-requiring 1*α* (IRE1*α*), double-stranded RNA-dependent protein kinase- (PKR-) like ER kinase (PERK), and activating transcription factor 6 (ATF6), which have been comprehensively reviewed [2, 3]. The UPR signaling, mediated through ER stress sensors, modulates transcriptional and translation programs in cells under ER stress. As a double-edged sword, the UPR provides survival signals at the initial phase of stress response, leading to cell adaption to ER stress [1, 2, 4]. When ER stress gets prolonged, the UPR can induce cell death programs to kill the stressed cells. In recent years, the scope and consequence of ER stress and UPR have been significantly expanded. Many pathophysiologic stimuli, such as oxidative stress, proinflammatory stimuli, fatty acids, and energy fluctuations, can directly or indirectly cause ER stress and the UPR activation in specialized cell types, such as macrophages, hepatocytes, and pancreatic *β* cells [2, 5]. The UPR signaling is fundamental to the initiation and progress of a variety of diseases, including metabolic disease, cancer, cardiovascular disease, and neurodegenerative disease [2, 6, 7].

## 2. LD Formation

LD, also known as adiposome or fat body, has been found ubiquitously present in lipid-overloaded cells from yeast to mammals [8, 9]. For a long time, LD was thought simply as an inert lipid storage reservoir since its earliest description in 19th century. The discovery of perilipin, an LD-associated protein that coats LD in adipocytes, makes researchers to challenge the understanding of LD as lipid storage [10]. LD is now recognized as a dynamic organelle composed of a monolayer phospholipid, embedded with numerous proteins without transmembrane spanning domains, and a hydrophobic core that contains triacylglycerols (TGs) and sterol esters [11, 12]. TGs are the key neutral lipid required for LDs formation in adipocytes. Deletion of genes encoding enzymes responsible for neutral lipid synthesis eliminated LDs formation [13]. Evidence showed that, without DGAT enzymes, LDs cannot form in adipocytes. Therefore, by segregation of extra TG or hydrophobic molecules into LDs, cells are protected from lipotoxicity. These features make LD a regulatory organelle in lipid homeostasis. The biogenesis and assembly of LD are still largely unknown. It has been suggested that ER is the site where LD is synthesized and assembled. Over ninety percent of LDs were found in close apposition to the ER [14]. ER budding model, Bicelle model, and vesicular budding model have been suggested to explain how LD is formed in ER [15]. Perhaps, the most accepted model is ER budding model in which LD originated between the two leaflets of ER bilayer buds into the cytosol. Newly formed LD can increase its size (0.2 *μ*m–20 *μ*m in diameter) by homotypic fusion that depends on microtubule system, most likely motor protein dynein. Under this mechanism, the growth of LD may proceed without ongoing biosynthesis of TGs and sterol esters [16, 17].

## 3. ER Stress and LD Formation

**Figure 1:**
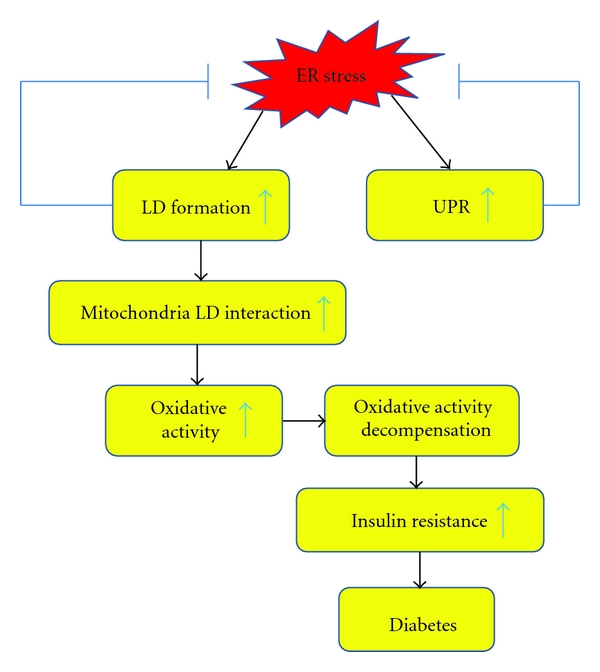
Interactions between ER stress, oxidative stress, and lipid droplets in type II diabetes. LD, lipid droplet; UPR, unfolded protein response.

LD formation has been proposed as an exit model in the removal of unfolded or misfolded proteins or some ubiquitinated proteins from the ER [18, 19]. LD may serve as a transient depot to sequester unfolded or misfolded as well as excessive proteins to alleviate ER stress (Figure 1). Diverse groups of LD-associated proteins were found in yeast *S. cerevisiae*, *Drosophila* embryos, and human hepatocyte cell line Huh7 [20–22]. Some of the LD-associated proteins, such as Acl-CoA synthetases, lanosterol synthetase, and GAPDH, are conserved from yeast to human. The proteins detected in LD seem to be specific, since the organelle-specific proteins, including lactate dehydrogenase (LDH) (cytosolic marker), integrin (plasma membrane marker), calnexin (ER marker), and GS28 (Golgi marker), were hardly detected in LD fractions [22]. Interestingly, a number of proteins which were thought to be organelle-specific, including histones (nucleus), caveolins (plasma membrane), HSP70 (cytosol), ApoB (ER), and Nir2 (Golgi), were detected in LD fraction [23]. Furthermore, LD dynamically interacts with ER, peroxisomes, mitochondria, and plasma membrane [15]. LD can be transported along microtubules, following the same way that the ER, Golgi, and mitochondria were positioned and delivered [24]. It was proposed that the dynamical interactions between LD and the other compartments facilitate the exchange of proteins and lipids in cells. The LD is functionally and structurally similar to the extracellular counterpart of lipoprotein particles [15, 21]. This notion was supported by the finding that LD provides a platform for degradation of excessive ApoB protein by converging ubiquitin-proteasomal and autophagy-lysosomal pathways, thereby preventing cytotoxicity resulted from aggregation of excessive proteins [25]. Previous studies have shown that disruption of ER functions leads to the accumulation of intracellular lipids [26–28]. Disrupted protein glycosylation or ER-associated protein degradation by ER stress-inducing reagents, such as tunicamycin and brefeldin, has been demonstrated to increase LD accumulation in budding yeast *Saccharomyces* cerevisiae or mammalian cells [28, 29]. Previously, it is known that intracellular LD formation is through the lipogenic program activated by sterol regulatory element-binding proteins (SREBPs). Recent study suggested that more ER-localized, stress-responsive protein factors, such as hepatocyte-specific cAMP responsive element-binding protein (CREBH), can also regulate lipogenic programs to promote LD formation under metabolic stress signals, such as insulin and saturated fatty acids [30]. Moreover, ER stress response may directly facilitate LD synthesis and assembly as a mechanism to defend intracellular stress [29, 31] (Figure 1). This is consistent with the observations that lipids can be recruited to the stressed cells to sequester misfolded proteins in the ER at the early stage of ER stress and that the ER is expanded significantly to alleviated ER stress independent of the UPR [23, 32].

## 4. LD Formation and Type II Diabetes

Previous studies demonstrated that excessive accumulation of lipids in peripheral tissues is closely associated with insulin resistance in type II diabetes [33, 34]. Although ER stress and UPR pathways in metabolic disease have been extensively reviewed, ER stress-associated LD formation, which is independent of UPR pathway, did not draw much attention. The interaction between LD and mitochondrial might affect the peripheral tissue insulin resistance [35, 36] (Figure 1). Recent studies indicated that insulin resistance is not simply associated with the amount of intracellular lipids. Despite elevated lipids content in skeletal muscle of the trained enduring athletes, the insulin-signal in these individuals is still markedly sensitive [36]. The combination of weight loss and physical activity in obesity improves insulin sensitivity and reduces the size of LD, but not the overall intramyocellular lipid [37]. One possible explanation for these phenomena is that increased mitochondrial oxidative activity for lipid oxidation may decrease insulin resistance. This is supported by the facts that lower oxidative capacity is found in insulin resistant skeletal muscle and that exercise can improve the capacity for lipid oxidation [36]. Several mitochondrial proteins including prohibitin, a subunit of ATP synthase, and pyruvate carboxylase were identified in LD fractions by proteomic analysis [35]. In addition, numerous lipid metabolic enzymes, such as hormone-sensitive lipase, lanosterol synthase, and acyl-CoA synthetase, were also found to be associated with LD complex, and the overall LD protein composition can be changed in response to lipolysis stimulation [35, 38]. Despite these observations, further study is required to explore how mitochondria communicate and interact with LD in metabolic processes.

Fat-specific protein 27 (Fsp27) is a member of cell death-inducing DNA fragmentation factor family proteins that is localized to LD. Fsp27 plays an important role in lipid storage and mitochondrial activity in adipocytes [39–41]. Genetic depletion of Fsp27 in mice is characterized by increased glucose uptake, improved insulin sensitivity, and significantly increased mitochondrial metabolism [39, 40]. Small sizes of LDs and increased mitochondrial activity were found in Fsp27-deficient white adipocytes, suggesting that ectopic LD formation represents an imbalance between lipid supply and lipid oxidation in peripheral tissue. Likely, LD-associated proteins and the interactions between LD and the other intracellular organelles may play direct roles in the pathogenesis of diabetes [42]. Type II diabetes is often correlated with increased serum levels of proinflammatory cytokines secreted by ER stress-activated macrophage. Previous research demonstrated that the proinflammatory cytokine TNF*α* blunts the insulin signaling pathway therefore causing insulin resistance by activating the JNK1/2 signaling pathway which is involved in serine phosphorylation of IRS1 (insulin receptor substrate 1) [43, 44]. However, a new study by Ranjit found that proinflammatory cytokines, such as TNF*α*, IL1*β*, and INF*γ*, act on lipolysis by decreasing the expression of FSP27 and the size of LD in adipocytes [45]. Since decreased FSP27 is evidenced to improve insulin resistance and LDs, it is likely that the proinflammatory cytokines play double-edged roles in type II diabetes.

## 5. Conclusion

Accumulating evidence demonstrated a strong link between ER stress, LD formation, and type II diabetes. It is important to note that ER stress response is a fundamental stress signaling underlying many life styles, such as air pollution, chronic alcohol consumption, and smoking, which may be associated with the development of metabolic disease [46–48]. Therefore, for the future research, it is important to delineate ER mechanisms in LD formation that is associated with the development of type II diabetes. Key questions include what is the mechanism by which ER stress regulates LD formation? Is there any ER chaperones or UPR targets present in the LD complex? Does ER stress-associated LD formation provide survival or devastating pathways in the progression of type II diabetes? Is it possible to modulate LD formation by targeting ER stress signaling? Answering these questions will benefit and direct the future understanding and treatment of type II diabetes and the other types of metabolic disease.
